# How scientists can contribute to the social movements essential to protecting climate and nature

**DOI:** 10.1038/s44168-025-00268-9

**Published:** 2025-07-31

**Authors:** Abigail J. Perrin, Stuart Capstick, Tracey Elliott, Peter Knapp, Aaron Thierry, Tristram D. Wyatt, Charlie J. Gardner

**Affiliations:** 1 Department of Biology, University of York, York, UK; 2 School of Psychology and Centre for Climate Change and Social Transformations, Cardiff University, Cardiff, UK; 3 Independent consultant, London, UK; 4 Department of Civil and Environmental Engineering, Imperial College London, London, UK; 5 School of Social Sciences, Cardiff University, Cardiff, UK; 6 Department of Biology, University of Oxford, Oxford, UK; 7 Centre for Biodiversity and Environmental Research, University College London, London, UK; 8 Durrell Institute of Conservation and Ecology, University of Kent, Canterbury, UK

**Keywords:** Ecology, Climate sciences, Environmental sciences, Environmental social sciences, Scientific community, Social sciences

## Abstract

Scientists have much to contribute to the growing social movements pushing for urgent and transformative change to address the climate and biodiversity crises. Depending on their skills, interests and circumstances, scientists can actively participate in social movements as members (whether on the streets or behind-the-scenes), endorse and facilitate these movements in their professional capacity and within their institutions, and build social movement effectiveness through research and teaching.

On climate and nature, the world is still heading in the wrong direction, and fast: greenhouse gas emissions continue to rise, the planet is getting hotter, extreme weather events are becoming more frequent, and the destruction of biodiversity continues apace^1^. This rapid change in planetary conditions is already having devastating impacts on human societies, yet governments worldwide continue to ignore scientific warnings calling for urgent and transformative change, choosing instead to implement policies in favour of vested interests^2,3^. In short, social and political systems at all levels are failing the planet, despite the best efforts of scientists to call attention to the “rapidly closing window of opportunity to secure a liveable and sustainable future for all”^4^. As scientists’ best efforts fail to move governments, we face a dilemma in which traditional expectations of professional detachment do not sit easily alongside the enormity of the climate and ecological crisis^5^. Here, we explore the practical ways scientists across all disciplines, backgrounds and career stages can be involved in activism^6^.

In response to the systemic failure of governments to act on the scientific evidence, over the past 5 years a new wave of environmental social movements has been actively challenging the balance of power and elevating these crises up the public, media and political agenda. These movements provide vital opportunities to push for change and we argue there are myriad ways for the science community to support them. As empiricists, we can take encouragement from the fact that the Intergovernmental Panel on Climate Change itself recognises, with ‘high confidence’, that “collective action as part of social or lifestyle movements underpins system change”.

From school strikes and Extinction Rebellion to the ecosystem of new groups emerging across the world, these organisations use wide-ranging approaches from traditional campaigning and protest to direct action and non-violent civil disobedience (NVCD), to exert political pressure on decision-makers to be more ambitious and draw public and media attention to the climate and ecological emergency. In recent years, climate activists have targeted fossil fuel companies, government departments, banks funding fossil fuel companies, media organisations and others responsible for driving environmental disasters or obstructing remedial measures; tactics have included occupying buildings, blocking infrastructure, interrupting meetings, and disrupting public appearances of key protagonists. Yet, to be truly effective as a force for transformative change, these social movements need to grow rapidly in size, reach and impact, all of which we argue can be enhanced by greater support and participation from a broad range of scientists^6^.

As scientists, our professional ethic requires us to be led by the best available evidence, which on climate and nature points to the need for far-reaching and urgent action. But it is important not just to express concerns verbally and in writing but to act in ways commensurate with our warnings. In doing so, our message rings true and can hit home^7^. In not doing so, we risk undermining our own advice; after all, policy-makers and the wider public are entitled to ask how serious these problems really are if scientists are conspicuous by their absence within those social movements pushing for change. With these motivations in mind, in recent years there has been increasing activism by scientists, including the creation of groups such as Scientists for Future, Scientists for Extinction Rebellion, and Scientist Rebellion (active in over 30 countries)^8^. The scientists involved in these and other groups have consciously crossed the invisible line that separates the calm of the lab from the noise of the streets, to affirm the reality and urgency of the crises we face. While protest and civil disobedience are not the only ways to participate, several recent papers make the case that where this is undertaken by scientists it can be particularly powerful (though see ref. ^9^ for considerations regarding effectiveness) because they—we—are still widely trusted and respected messengers^7,10,11^. Being involved in actions or advocacy does not seem to impact deleteriously the public’s trust in scientists^9^ and there is a strong case that scientists should not be ‘neutral’ on issues such as the climate and biodiversity crises^3,12,13^.

Globally, however, there remains a wide discrepancy between the large numbers of scientists stating they are willing to engage in climate advocacy (58%), participate in legal protests (47%) or take part in NVCD (45%), and the much lower numbers already engaged in these activities (29%, 23% and 10%, respectively)^14^. In other words, many scientists have indicated they are willing to join social movements but are not currently doing so in practice. This may partly be influenced by recent political crackdowns on protest in many countries, which particularly limits involvement by those who face additional systemic barriers to participation, such as scientists of colour or other discriminated-against groups, those working in less-tolerant countries or on precarious contracts, or those with caring responsibilities. However, even those scientists who are willing to become more involved in any form of climate advocacy have reported barriers and concerns^14,15^ including: the perception that they lack the required skills; fear, even if unwarranted, of losing credibility, public trust and/or professional reputation; impact on employment prospects; a lack of time, opportunities or institutional support to engage^16^; experiences of anxiety or burnout, especially given the heavy emotional toll that can accompany engagement with the realities of the climate and biodiversity crisis^17^; or simply not knowing “how to get started”^18^. Here, we outline how scientists can contribute to social movements both in a professional capacity and as global citizens, and depending on their particular skills, interests and circumstances (Fig. 1).

**Fig. 1:**
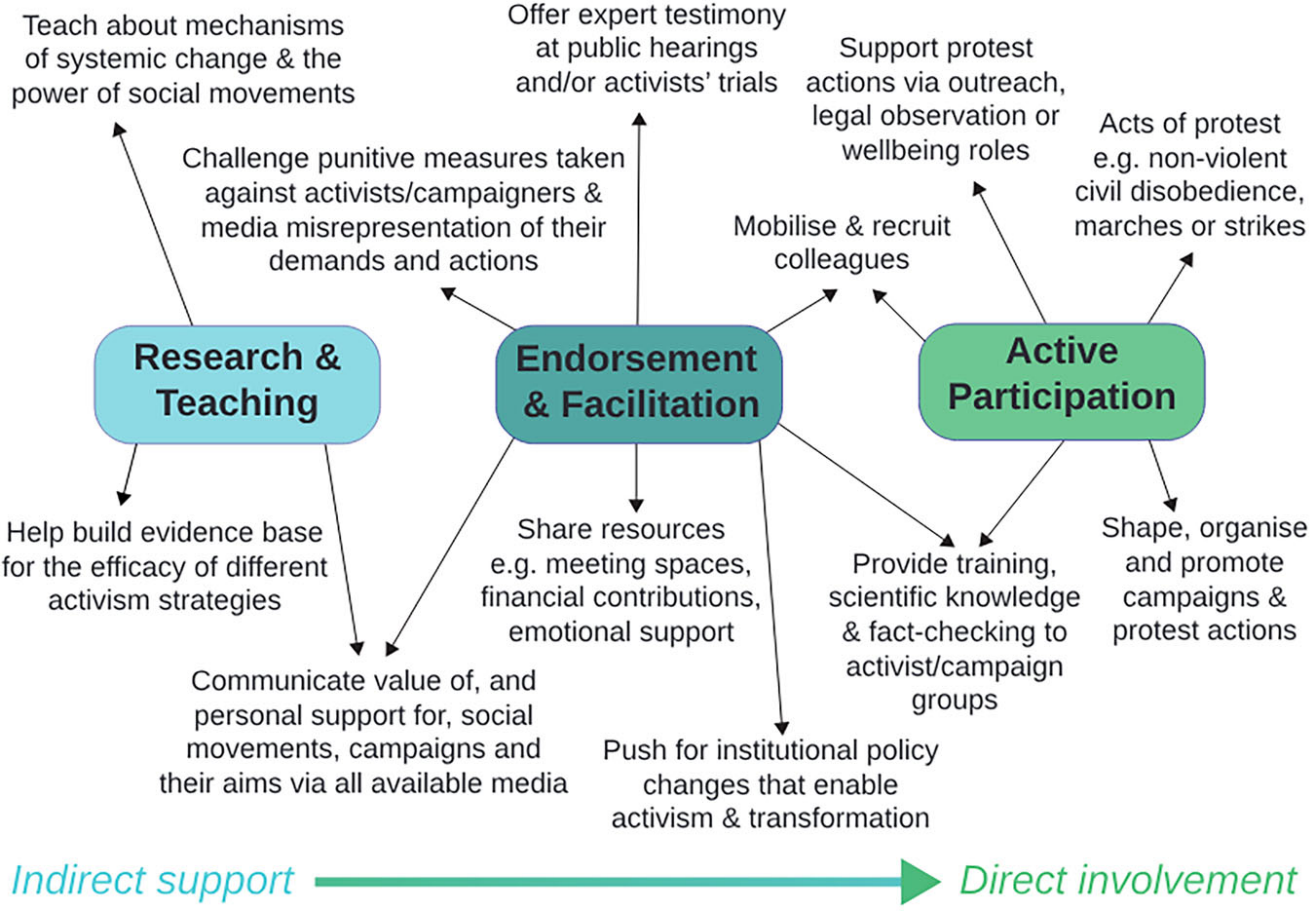
How scientists can support social movements: an overview of different actions across a spectrum from indirect assistance to direct participation.

## Research and teaching

Although there is a body of research on social movements, such as the Civil Rights movement in the US, historical analogies can only be taken so far: modern environmental and climate social movements face an unprecedented challenge and are operating with a limited evidence base as to how they can maximise their influence and effectiveness. Researchers with relevant skills can help generate the knowledge required^19^, by refocusing their research to investigate, for example, the effectiveness of different activism strategies and targets in a range of contexts^20,21^. This could include the use of participatory research and citizen science^22^ as well as experimental studies of tactics and messaging. Scientists who teach can additionally seek to educate their students about social movements, by incorporating material about how social change happens into their curricula^23^. Closer engagement with social movements allows for greater co-production of knowledge and the development of a more horizontal and collaborative relationship between science and society^13^.

## Endorsement and facilitation

While they don’t necessarily strive—or expect—to be popular with everyone, social movement participants do generally aim to influence public opinion constructively^24^. However, they face a media environment that regularly seeks to trivialise, undermine, and demonise them. In such a context, voices of support offer a powerful counter-narrative, particularly when they come from respected and trusted citizens such as scientists. Whether through scientific publications, op-eds or letters to the editor, media interviews, social media, or simply in private conversation, vocal support from the scientific community confers credibility and legitimacy to social movements.

Scientists can offer support as experts with knowledge of the seriousness posed by multiple environmental crises. For example, academics have been able to support activist campaigns against destructive infrastructure development by speaking at public hearings, as well as providing expert witness testimonies for activists in court for acts of civil disobedience. Such support may be the most effective way to ensure that decision-makers, judges and juries are fully aware of the scientific rationales underpinning the demands and actions of activists. In addition, scientists can offer material support directly to social movements, including by shedding light on complex or inaccessible research, or offering talks or training to ensure activists are well informed. For example, Scientists for Future have compiled open letters^25^ and fact-sheets to help the climate movement communicate the severity of the situation we are in.

Scientific and higher education institutions are themselves important sites of engagement for the climate movement^21^. Reforming campaigns (often led by students) are currently pushing for divestment of university finances from polluting industries^26^ or cutting other research ties to them, for example by preventing fossil fuel industry representatives from sitting on governance boards^27^. There are also efforts to decarbonise laboratories and campuses^21^, and to phase meat out of the menus of university canteens as part of a shift to sustainable diets^28^. As engaged members of these communities, researchers can assist such efforts by backing them. Scientists can also work for cultural and policy change within our own institutions, including research institutions, science academies and professional bodies. This can include lobbying for institutions to make formal declarations of support for social movements such as in the declaration of a climate emergency^29^, recognising the right of staff to take part in nonviolent forms of protest without risk of professional censure, and recognising participation in advocacy and activism as part of the core mandate (and ultimately evaluation) of academics.

## Active participation

Some environmental social movement organisations carry out disruptive actions that carry a risk of arrest, which can achieve significant public attention via social media and press coverage (e.g., ref. ^30^). Scientists can shape, organise and promote such actions, or participate directly and risk arrest: renowned scientists to have placed themselves in this situation include Carl Sagan, arrested for crossing security barricades in an act of civil disobedience while protesting nuclear weapons testing; James Hansen, who has been arrested multiple times opposing fossil fuel infrastructure, including for his part in sit-in protests outside the White House; and Julia Steinberger, arrested in Switzerland at a climate protest for obstruction of traffic. However, these actions themselves are merely the tip of the iceberg when it comes to the effort involved by active members in making such groups effective. Much other work takes place away from the public eye, and consists of organising meetings, recruiting and training new members, communicating through press and social media, developing strategy, planning actions, liaising with partners, fundraising, and enhancing the collective capacity of the organisation. Notably, much of the work of social movements requires the types of professional skills that scientists have spent their careers developing, including how to retrieve, process and critically analyse information, how to communicate effectively to a range of audiences, how to work within teams and how to plan and implement projects. Less science-related contributions include everything from making banners and other artworks to offering emotional support to other activists.

Scientists can also play a supporting role in acts of civil disobedience with only minimal risk of arrest, for example by acting as legal observers, police liaison or spokespeople, offering welfare or de-escalation support, or by speaking to passers-by about the actions. Social movements also use a range of tactics beyond disruptive civil disobedience, many of which provide routes for scientists to take part in acts of protest that may be perceived as less risky or controversial. These include boycotts, strikes and walk-outs, street theatre, mock award ceremonies, teach-ins, and fasting.

Although traditional scientific approaches to communication have failed to sufficiently influence policy to date, scientists have a huge, but largely untapped, potential to accelerate progress; by stepping beyond our traditional roles to support or participate in social movements, or by embracing advocacy and activism as an integral part of our roles^5^, scientists can help catalyse the transformative changes we urgently need^3,10,23^. Since the impact and ultimate success of these movements depend in large part on the numbers of people who rally behind them, the engagement of scientists can confer further legitimacy and confidence in them, and thus help with recruiting more support. In providing a range of ways in which scientists can engage, with different levels of personal risk, we urge readers to think hard about how our knowledge, skills, positions and influence can be most effectively channelled to help steer us towards that liveable, sustainable future for all. If not now, when?
